# Extreme, Cold‐Season Climatic Events Can Decimate Wildlife and Imperil Population Persistence

**DOI:** 10.1111/gcb.70772

**Published:** 2026-02-24

**Authors:** Adele K. Reinking, Katherine B. Gura

**Affiliations:** ^1^ Cooperative Institute for Research in the Atmosphere Colorado State University Fort Collins Colorado USA; ^2^ Graduate Degree Program in Ecology Colorado State University Fort Collins Colorado USA; ^3^ Department of Fish, Wildlife, and Conservation Biology Colorado State University Fort Collins Colorado USA

The coalescent processes that define ecological systems, such as population dynamics, community structure, and species composition, can be fundamentally modified by environmental changes. These changes range from gradual, predictable gradations to extreme, stochastic events. Extreme climatic events are, by definition, rare and substantial environmental perturbations. However, under global climate change, they are becoming more frequent and severe (Ummenhofer and Meehl 2017). These phenomena occur in a wide array of ecological systems and take many forms (e.g., wildfires, floods, heat waves, hurricanes, ice storms). Despite the growing pervasiveness of extreme climatic events, their long‐term ecological effects are poorly understood and remain a critical uncertainty when predicting vulnerability to climate change (Ummenhofer and Meehl 2017; Maxwell et al. 2019).

Climate regions characterized by seasonal or perennial snow (i.e., Arctic, Subarctic, Continental, Temperate) are undergoing some of the planet's most drastic changes due to rapid, high‐latitude warming. For example, climate change is altering the timing, frequency, and intensity of storm cycles and rain‐on‐snow events; the means, maxima, and minima of daily temperature and snowfall; and wind regime dynamics (Hansen et al. 2014; Ummenhofer and Meehl 2017; Maxwell et al. 2019). Resultant extreme climatic events are emerging as a potentially devastating source of mortality for wildlife experiencing snow and cold‐weather processes, and multiple extreme climatic events in succession can compound one another to be especially disastrous. For example, in 2020, a sudden winter cold snap, following a malnutrition‐inducing summer of severe drought and wildfires, resulted in a mass mortality event for hundreds of thousands of migratory passerines across the southwestern United States (Irannezhad et al. 2022). Extreme cold‐weather events have proven similarly detrimental for ungulate populations. A record‐breaking snow season in the western United States during the winter of 2022–2023 caused a 3.7‐fold increase in mortality rates for pronghorn ( *Antilocapra americana* ) in Wyoming, USA (Aikens et al. 2025). Likewise, an extreme rain‐on‐snow event during the winter of 2011–2012 in Svalbard, Norway created extensive, thick ground‐icing that precipitated widespread starvation of reindeer ( *Rangifer tarandus platyrhynchus* ) (Hansen et al. 2014). Finally, avalanches, which are powerful and destructive events in which snow abruptly releases and flows downslope (Eckert et al. 2024), have emerged as a significant source of mortality for mountain goats ( *Oreamnos americanus* ) in coastal Alaska, USA (White et al. 2024).

Although recent research has illuminated many immediate effects of extreme climatic events on individual survival, knowledge of how stochastic conditions influence protracted demographic responses has proven more elusive (Maxwell et al. 2019), especially in snowy environments. Concurrent, long‐term monitoring of both populations and environmental conditions is inherently challenging in many snow‐covered regions because of their remoteness, harsh conditions, and possibly high topographic variability. The difficulty of quantifying snow properties in situ across space and time, especially in remote environments, impedes the ability to track historic trends, understand spatiotemporal variation in current conditions, or predict future snow patterns with confidence (Revuelto et al. 2025). Usually, ecologically relevant snow information covering the areas and time periods of interest at the ideal spatiotemporal resolution is simply not available. Similarly, extensive and detailed wildlife monitoring datasets that closely track many individuals and span generations require significant, sustained resource investment and are, therefore, rare. These shortcomings limit our capacity to gain new insights by mechanistically linking wildlife and cold‐weather condition data (Reinking et al. 2022). Long‐standing studies that contextualize stochastic, extreme climatic events within broader climate patterns, and relate these events to population dynamics, remain critical to adequately understand demographic responses and resilience to climate change.

A new Research Article published by White et al. (2025) in *Global Change Biology* provides a quantitative modeling framework to evaluate how extreme climatic events influence population dynamics of a long‐lived ungulate species occupying remote, snowy environments. White and colleagues paired a sex‐ and age‐structured population model, based on a robust, 44‐year mountain goat monitoring dataset, with cause‐specific mortality information to simulate the effects of avalanche‐related mortality on mountain goat population trajectories. Building upon initial findings of the surprisingly dominant role of avalanches in directly causing coastal Alaskan mountain goat deaths (White et al. 2024), likely through physical trauma during the slide or asphyxiation after burial under snow, White et al. (2025) evaluated how variability in observed rates of annual, avalanche‐related mortality influenced population growth, viability, and recovery time. Across both short (2‐year) and long (30‐year) time scales, annual, avalanche‐related mortality rates significantly influenced annual population growth. Mountain goat populations were estimated to increase moderately if no animals died in avalanches, increase only marginally under current, average avalanche mortality conditions (i.e., 7% of population killed by avalanches annually—considered compensatory mortality), and decline under severe avalanche mortality (i.e., 23% of population killed by avalanches annually—considered additive mortality). Under the most severe, annual, avalanche‐caused mortality rate of 23%, estimated population recovery time was 11.2 years, or roughly 1.5 times one mountain goat generation. Under a slightly lower annual, avalanche‐caused mortality rate of 19%, population recovery was estimated to require 7.2 years, or one mountain goat generation. This study's findings indicate that severe avalanche conditions, resulting in the highest observed annual mortality rates, are likely to produce significant population declines via additive mortality and require extensive population recovery times. Additional extreme climatic events, including non‐avalanche disturbances, occurring within those recovery periods would further exacerbate the demographic impacts, jeopardizing long‐term population persistence.

Although climate change studies have primarily focused on shifting means of environmental conditions, or chronic, unidirectional change (e.g., steady temperature increase), there is mounting evidence that extreme climatic events may have greater potential to create significant, long‐lasting consequences for ecological systems (Maxwell et al. 2019). White et al. (2025) utilized a rare, long‐term, population‐monitoring dataset to address questions about demographic consequences of such stochastic disturbances. These questions are uncommonly answered but increasingly pressing under current environmental change. A particularly salient component of this research is that many extreme climatic events, including avalanches, equally impact individuals with varying life history traits (e.g., age class, life stage, reproductive status), rather than disproportionately acting on the most vulnerable individuals within a population (White et al. 2024, 2025). Climate change is generally expected to increase the proportion of wet relative to dry avalanches (except at lower elevations, where snowpacks are typically declining), but information on future trends in avalanche frequency and magnitude remains sparse (Eckert et al. 2024). More avalanche activity could result in the precipitous decline of relatively small, isolated populations of species such as mountain goats or others inhabiting similar environments (e.g., Dall's sheep [ *Ovis dalli* ]). Such population losses have the potential to cause cascading effects on community and ecosystem dynamics, including plant biodiversity, predator–prey relationships, and nutrient cycling (Ummenhofer and Meehl 2017). More broadly, this research casts disturbing prospects for many different ecological systems and their inhabitant wildlife communities experiencing increasingly severe, stochastic weather and climate conditions.

White et al. (2025) highlight the critical need for datasets that not only facilitate holistic, mechanistic understandings in rapidly changing systems and populations, but also allow their contextualization over broader spatial domains, historically and into the future. Because of the paucity of information regarding historic and current avalanche regimes, White et al. (2025) were unable to directly investigate how avalanche frequency and severity have changed through time in their study areas. Nor did they explore relative effects of different avalanche types (e.g., wet vs. dry, slab vs. point release, caused by new snowfall vs. wind‐loading vs. recent melt) that could aid understanding of the precise conditions posing the greatest threat to mountain goats, or how the frequency and severity of those particular conditions might be changing (Eckert et al. 2024). Improved local assessments, long‐term monitoring, and weather and snow models are required to better understand climate change impacts on avalanche conditions and the ecological outcomes of these disruptive events (Reinking et al. 2022; Eckert et al. 2024). Such information would enhance knowledge regarding how avalanche‐mountain goat relationships have changed over time, and perhaps more critically, could facilitate quantitative prediction of their character into the future.

Promising avenues to expand weather and snow data coverage, quality, and ecological relevance, such as the merging of point observations, remote sensing, and modeling tools (i.e., “data‐model fusion”) to produce spatiotemporally continuous weather and snow data, are a burgeoning area of interest (Reinking et al. 2022; Revuelto et al. 2025). Similar datasets and modeling systems across other climate regions are equally critical. For example, improved flood prediction tools or more comprehensive characterization of the range of possible temperatures in a system (accounting for extreme heat or cold waves) could elucidate exposure and sensitivity to these climatic events, two critical metrics in formal vulnerability assessments for species and communities of conservation concern (Maxwell et al. 2019). With such information in‐hand, the opportunities for groundbreaking research and novel insights into extreme climatic events and their demographic ramifications for wildlife are multiplied. The recent work of White and colleagues exemplifies the power of robust, long‐term datasets for advancing our understanding of population vulnerability in the face of global climate change and presents a framework for analyzing these relationships, when and where the required supporting data exist.

## Author Contributions

**Adele K. Reinking:** supervision, project administration, conceptualization, writing – review and editing, funding acquisition. **Katherine B. Gura:** writing – original draft preparation, writing – review and editing, funding acquisition.

## Funding

This work was supported by the National Science Foundation, 2402348.

## Conflicts of Interest

The authors declare no conflicts of interest.

## Data Availability

The authors have nothing to report.
